# Psychological distance: a qualitative study of screening barriers among first-degree relatives of colorectal cancer patients

**DOI:** 10.1186/s12889-021-10786-w

**Published:** 2021-04-13

**Authors:** Xueying Zhang, Yiheng Zhang, Jingyu Chen, Meifen Zhang, Ni Gong

**Affiliations:** 1 School of Nursing, Sun Yat-sen University, No. 74 Zhongshan 2nd Road, Guangzhou, 510080 Guangdong China; 2 School of Nursing, Jinan University, No.601 West Huangpu Avenue, Guangzhou, 510632 Guangdong China

**Keywords:** Colorectal cancer, Screening barriers, First degree relatives, Medical technology, Qualitative research

## Abstract

**Background:**

Colorectal cancer screening can reduce the incidence and mortality through early detection. First-degree relatives (FDRs) of patients with colorectal cancer are at high risk for colorectal cancer and therefore require colonoscopy. However, despite the high risk, screening adherence among FDRs remains low and the barriers to undergoing screening among FDRs in China are not clear. We explored the reasons why FDRs refused screening.

**Methods:**

In this qualitative study, 28 semistructured, in-depth interviews were conducted face-to-face. Participants were recruited at two hospitals (an urban tertiary hospital and a community health center) in Guangzhou, South China. We used qualitative content analysis to analyze transcripts based on audio recordings and identify major themes and subthemes.

**Results:**

Three major themes emerged related to FDRs’ low screening participation. First, the emotional distance between FDRs and medicine was pulled away by uncomfortable feelings approaching hospitals and misunderstanding of cancer. Second, they confirmed their health state and minimized cancer risk if they had no signs in routine health examination, no symptoms and maintained a healthy, happy life. Third, they considered screening far from their daily life from the perspective of spatial distance and priority. Therefore, screening was not necessary in their view.

**Conclusions:**

Healthcare professionals should narrow psychological distance between people and screening when promoting screening technology.

**Supplementary Information:**

The online version contains supplementary material available at 10.1186/s12889-021-10786-w.

## Background

Colorectal cancer (CRC) is the third most common malignant tumor with the second highest mortality rate in the world [1, 2]. According to GLOBOCAN 2018, there were 1,800,000 new cases of CRC and 88,000 deaths, corresponding to 10.2% of new cases and 9.2% of all cancer deaths, respectively [1]. The early cure rate of CRC is > 90% [3], while the 5-year survival rate of patients with advanced treatment is < 10% [4]. Unfortunately, 17.6–35.1% of patients are diagnosed at an advanced stage [5]. Early diagnosis and screening can greatly increase the early detection rate [6], thereby improving the therapeutic effect [3], reducing morbidity and mortality [7], and saving treatment costs and health resources [8].

First-degree relatives (FDRs) are almost two or three times more likely to develop cancer than the general population at average risk [9, 10]. The guidelines recommend FDRs should be screened earlier than the average risk adults [11, 12]. According to China Guideline for the Screening, Early Detection and Early Treatment of Colorectal Cancer (2020, Beijing) [13], FDRs should start to be screened at 40 years old, or 10 years younger than the age at which the youngest affected relative was diagnosed with CRC. However, the screening rate of FDRs around the world remains very low, at 22.2 to 65.8% [14, 15]. According to a study in National Clinical Research Center for Cancer in China, only 20.9% participants underwent colonoscopy as recommended [16]. One of the criteria for an effective screening program is that over 70% of the target population participate in the screening [17]. The phenomenon constitutes the core issue of this study: Why does cancer screening, which theoretically benefits FDRs, serve as a barrier in practice?

Previous studies have explained why FDRs refuse screening from several perspectives. First, due to the lack of knowledge on disease severity and susceptibility and screening effectiveness and modalities [18], FDRs may not think screening matters [19]. Second, some people equated cancer with death [20, 21], making them fear of cancer and worry about high surgical costs even when just considering being screened. At the same time, current screening techniques also cause discomfort such as pain and embarrassment associated with bowel preparation before colonoscopy [22]. Numerous studies have shown that screening behaviors are affected by education level, health literacy, health insurance, healthcare professional recommendation, family supervision, and perceived barriers of screening [18, 23, 24].

Previous studies have suggested that screening barriers among FDRs are due to poor understanding and acceptance of screening [21, 25, 26]. However, existing research attaches importance to individual direct experience, which ignores social environment, the profound impact of cultural situations on individuals. Furthermore, most studies were undertaken in Western countries including the United States and Europe [22–25], with few in developed Asian countries [21, 27], especially in China [18]. Finally, despite the fact that most screening processes and promotions takes place within hospitals, FDRs’ daily life might be changed as a result of screening. Therefore, the psychological distance between individuals’ daily life and medical technology should be taken into account.

Psychological distance takes “oneself” as the center or a point of reference, and include four dimensions (temporal distance, spatial distance, social distance, or hypotheticality) [28]. According to construal level theory, psychological distance serves as a criterion by which an individual uses their past experiences as a basis to decide whether to make a decision at hand [28]. Prior studies have demonstrated how psychological distance affects individuals’ preferences for primary health care [29, 30]. A short psychological distance may make an individual execute specific goals, while they may postpone or not complete goals with long psychological distances. This may well explain the behavior of FDRs not participating in screening. For example, individuals’ preferences for genetic screening for CRC differed in their psychological distance especially in temporal and spatial distance within the Dutch national CRC screening program [30]. Nevertheless, there are few studies on how the psychological distance between the daily life of FDRs and screening technology influence their screening rate. Therefore, in this study, we used qualitative method to probe the perspectives of psychological distance between FDRs’ daily life and screening technology to identify factors that hinder screening among a high-risk population of FDRs of patients with CRC.

## Methods

### Design

This qualitative study recruited FDRs of colorectal cancer patients through purposive sampling. Data were collected between September 2017 and August 2019 at two hospitals (an urban tertiary hospital and a community health center) in Guangzhou, South China. Twenty-eight semistructured, in-depth interviews lasting from 36 to 77 min were collected face-to-face.

### Participants

Participants were eligible if they were parents, siblings, or children of patients who were diagnosed with CRC. Eligible participants aged 40–75 years old were included. Participants with mental illness or communication problems were excluded. In order to ensure the heterogeneity of the sample, we recruited not only the participants who had never done colonoscopy, but also the participants who had hesitated for a long time before colonoscopy. Participants’ thoughts during procrastination were also barriers to cancer screening. In addition, the experience of these participants could also help us understand why they ended up undergoing screening. We purposively recruited participants from the clinical center when they accompanied patients or they went to the community health center themselves. We also asked clinicians and head nurses to help identify and communicate with potential participants.

### Data collection

We finalized the interview questions (Additional File 1) after literature review, 3 interviews during the pilot phase and discussions with our team. Two researchers conducted the interviews in Mandarin after establishing relationships with participants prior to study commencement. All participants provided written informed consent for audio recording after we explained the research purpose and that participation was voluntary and confidential. The interviews took place in a relatively quiet, private, undisturbed room in researchers’ workplace (hospital and community). We clarified and recorded what they did not understand. Field notes were made to record non-verbal information during the interviews. We used maximum variation sampling to recruit a wide range of diverse participants [31]. No new information emerged in the last three interviews, indicating data saturation was reached, and no additional interviews were conducted.

### Data analysis

We analyzed the data through the process including open coding, creating categories, and abstraction [32]. The audio recordings were transcribed verbatim by two nursing students and verified by another student for accuracy. Two researchers independently and continuously reviewed and coded transcriptions of the recordings, using qualitative content analysis to generate themes and subthemes. Next, differences in coding were discussed by our team, including a nursing professor, a medical anthropologist, and three nursing students, to form categories until 100% agreement was reached. After this process, we found that the results could be explained by the concept of psychological distance [28]. In the context of psychological distance, the codes were categorized and regrouped. The final three themes were examined by the team to ensure the difference between them. All Chinese transcriptions were translated to English by a professional translator. In addition, one researcher compared Chinese and English versions sentence by sentence to ensure the accuracy of the translation and corrected grammatical errors. After the process of the content analysis, three themes and 10 subthemes were generated. Typical quotes from participants were chosen to support each theme and answer research questions. Participant’s names are replaced by numbers (e.g., P1) to ensure confidentiality.

## Results

With the help of clinicians and head nurse**s**, 30 potential participants were approached and two of them declined to be interviewed due to time inconvenience. The average age of 28 participants was 48.5 years. Of the 28 participants, 11 (39.3%) were female, and 7 (25.0%) underwent colonoscopy (Table 1). Three major themes emerged to describe the barriers to undergo screening: 1) Emotional distance from medical recognition; 2) Cognitive distance in health view; 3) Distance from daily life.

**Table 1:** Characteristics for the 28 participants

Characteristic	No. (%)
Age, Mean (SD), y	48.5 (9.0)
Sex	
Female	11 (39%)
Male	17 (61%)
Educational Level	
Did not complete high school	10 (36%)
< 4 y college	11 (39%)
College graduate or postgraduate degrees	7 (25%)
Had a colonoscopy in last 5 years	
No	21 (75%)
Yes	7 (25%)
Relation with colorectal cancer patients	
Children	20 (71%)
Sibling	6 (21%)
Parent	2 (7%)

### Emotional distance from medical recognition

Emotional distance refers to the emotional difference between the audience and the people or things involved in the content. For example, FDRs’ negative recognition towards cancer and hospitals has resulted in a far emotional distance between FDRs and medicine. These feelings include not only discomfort approaching hospitals, but also the misunderstanding that cancer is incurable and inevitability. Therefore, they will think of these bad emotions and are reluctant to participate in screening when hearing about cancer screening.

#### Negative feelings for hospitals

Some FDRs pronounced discomfort associated with hospitals, making it difficult for them to undergo screening. They often associate hospitals with sick or unpleasant things, which leads them to keep a long distance from hospitals in their life. Thus they tried to avoid contact with hospitals as much as possible in daily life, and even minimize travel to hospitals for non-essential cancer screening. Like participants said,

*I have developed a particular fear to go to the hospital since a family member of mine suffer from a medical accident, not to say let me go to the screening. (P23).*

*As long as I do not go to the hospital, I feel in good health. I try to avoid going to the hospital no matter whether it is because of my own problem or a friend’s problem, not to mention because of the cancer, which is likely not to happen. (P26).*

#### Misunderstanding of cancer

FDRs were amenable to considering whether to participate in screening. For FDRs, cancer was equal to terminal illness and death, and people often avoided discussing related topics. When asked why they did not get screening, one of our participants said:

*Developing any disease is acceptable to me, except for cancer, which is a terminal disease, very horrible, and getting it means basically incurable. I am very afraid of it. (P21)*

Cancer fatalism pushes screening away from people’s daily lives. Some FDRs believed that their cancer risk was not influenced by their own behavior, that everything was destined and cannot be evaded or changed, and that screening was not necessary in their lives if they were destined to have cancer. As one participant said,

*I resolutely refuse a screen, and this kind of thing depends on fate. If you are destined to have cancer, you will never change this fate! (P6)*

Recent developments in modern medicine have clarified the links between cancer and genetics, which further strengthens the sense of FDRs’ recognition of fatalism and distance towards screening. As one female participant said,

*This disease is genetically related, and there is no way to change, even for screening. (P15)*

### Cognitive distance in health view

Cognitive distance reflects the “potential difference” between individuals in terms of knowledge, resources, skills, etc. [33]. There was a cognitive distance in understanding of health and FDRs’ cancer risk between FDRs and clinical healthcare professionals. FDRs have developed their own concept of health through various means including no signs in routine physical examination, no symptoms, and a good life. FDRs’ understanding of health and diseases is complex and diverse which makes them be reluctant to accept screening recommendations.

#### Normal routine physical examination results equal health

Some FDRs regularly participate in routine health examinations and use data from medical instruments as criteria to define health. They believed that normal annual physical examination results meant good health and pulled them away from disease cognition, which made them feel no need for deliberate screening. But they did not realize that routine physical examination cannot completely replace cancer screening [34]. As one participant said,

*My work unit organizes a physical examination every year, and if a physical examination can detect any problem, why go to so much trouble to do a special examination? (P27)*

#### Absence of symptoms implies health

Cognition of disease was derived from the most direct physical experience, and they believed that the presence of symptoms such as pain and melena represented the possibility of CRC, and they equated the absence of symptoms with long distance towards disease. Therefore, they would not consider further testing or even screening in the absence of physical discomfort [35]. As a participant said,

*My body does not have any problem if it does not feel anything, and I do not have to go for screening. (P4)*

#### A good life means health

Some FDRs believed that health was not a simple disease-free state but also required a healthy and happy daily life. First, they believed they could avoid cancer risks and keep health as long as they actively took healthy lifestyle (e.g., regular life habits, healthy diet, active exercise, and maintaining a happy mood). With such perceptions, they thought they were far away from disease, so screening was unimportant. As the following participant said,

*The body needs to be nourished. At my age, it is insignificant to be screened...Living a full, happy, and regular life is better than treatment, examination, and taking medicine. (P17)*

At the same time, their longings for a better life included self-care, spending quality time with grandchildren, and extensive social activities. Based on their understanding, they believed that the current level of daily living had reached the standard of health and was far away from illness. Therefore, it was completely unnecessary to visit the hospital for screening.

*The most important thing for health is to be happy and fulfilled! It feels very good to be busy. I go to the park every morning to dance, then buy vegetables to cook, sometimes go to class in the afternoon, and often have activities in the evening. I am now fulfilled every day and living a healthy life without having to go for screening. (P24)*

### Distance from daily life

FDRs feel a sense of distance towards cancer screening within hospitals from the perspective of daily life. This sense of distance is reflected not only in physical space, but also in time and transportation cost. More importantly, screening is a lower priority in their daily life.

#### Spatial distance from the hospital

First, some FDRs found it difficult to receive screening independently and often required assistance from their children. In addition, they often lived far from the hospital, introducing problems such as time coordination and transportation to reach the screening site. As one of our participants said,

*Travelling to go for screening is very troublesome, and I need the companionship from my children. What makes it worse is that I cannot even use any public transportation, do not know the destination, so I choose not to come. (P9)*

Second, the cumbersome process of CRC screening felt bothersome and time consuming to FDRs, which in turn would increase their sense of distance from screening or even hospitals, so they delayed screening. As a participant said,

*It was too troublesome, I waited half a year before I received the painless colonoscope. But when thinking from another man's angle, he would not go to so much trouble visiting a hospital for so many times and then waiting. (P1)*

#### Maintain daily life prior to screening

Although some FDRs understood the importance of screening, their daily life was occupied by complex work, caring for parents, parenting, and other events or activities. They often had a sense of powerlessness. Therefore, screening was not a priority in their lives and was often postponed when matters concerning work or family appear simultaneously. This distance in priority could eventually lead to non-participation in screening.

*Because now things are more, the pressure is high, I need to pay mortgage for house and care, and my dad spends so much money here, I can't think about so much about screening! I also knows that screening is useful and that early detection of the disease will be better, but I have no time to go for it (screening).(P10)*

At the same time, the possible bad outcomes arising from screening can directly break the balance of the current “healthy” or even good life and immerse them in fear. Their daily lives would be completely controlled by the hospital, and they would need to fully live in a medically healthy way (including regular screening). Therefore, they were more likely to choose to maintain their current state of life than to be closed to unpredictable screening results.

*If this risk is confirmed by screening, I need to be worried all day long and go for reexamination at intervals. This feeling is bad. So instead of living with a good awareness of health but in pain, why not choose to take bliss in ignorance and live happily? (P3)*

## Discussion

FDRs, as a high risk population should begin early screening to timely detect and treat precancerous lesions to avoid progression to cancer. Our results suggested that even for FDRs who had been recommended for screening, their colonoscopy rate of 25% low compared with the effective screening rate of 70% [17]. In addition, we found that the psychological distance from screening developed by FDRs is an important reason that prevents them from undergoing screening. Therefore, this study specifically elaborates from three perspectives: emotional distance, distance from diseases, and distance from daily life.

First, FDRs’ sense of emotional distance limits their willingness to complete screening. Previous studies have found that FDRs have limited awareness of cancer [20, 21]. According to World Cancer Report, one third of cancers can be preventable, and another third can be cured. However, some people believe that cancer is an incurable disease, and the proportion is 43% in China [36]. They also have developed a stereotype that having cancer equals with terminal illness and death. They avoided to talk about related topics. Cancer screening, on the other hand, brought this taboo topic infinitely closer to their lives while they were trying to avoid it as much as possible [37]. This worsens the panic of FDRs about cancer, so there is resistance to cancer-related things including screening. As a fairly newly developed medical detection technology, many people in China do not understand the significance of screening for cancer prevention. In addition, some individuals feel whether they have cancer is controlled by fate [38, 39]. This perception makes individuals deny the significance and value of cancer screening, and the gap between cancer fatalism and prevention is difficult to bridge. Strong believers in the concept of fatalism are less willing to go for screening and more likely to delay seeking medical attention, even choosing to avoid screening [40]. In addition to physical space and time constraints, if FDRs have heard about cancer examination and treatment processes experienced by relatives and friends in the past, this will increase their psychological distance from the hospital, and they may subjectively reject colorectal cancer-related tests. The end of treatment of patients meant leaving the hospital, returning to daily life, and increasing distance from the disease and hospital for FDRs. However, screening requires returning to the hospital, and the superimposition of pain and displeasure in the hospital setting is the number one hinderance of screening.

Second, FDRs have a distant cognitive distance to the disease due to their own health perceptions and status. Individual health views differ from medical health. In health institutions, the determination of health is still focused on the biomedical model with the body as the core, and negative medical test results are judged to be reflective of a “health” status. However, FDRs’ understanding of health is derived from absence of symptoms. At the same time, the health perceptions of FDRs are not the same as that of medical discourse, which increases resistance to screening. In their daily life, they have different health concepts due to their unique life experiences [35], and their definitions of health vary from yearning for a disease-free life without suffering to the simple perception that absence of symptoms is health. This experience is consistent with findings from previous research among several countries, such as Korean [41, 42]. Therefore individuals measure their own health level according to their own perceptions, and when individuals believe they are in a healthy state with a small probability of getting sick, they develop a large cognitive distance from the disease [43]. It also reflects the effectiveness of communication intervention in promoting adherence rates [44]. Showing consideration of FDRs’ understanding of health formed in their daily life is an important part of effective clinical communication. This represents the second obstacle in which the sense of distance between health and disease hinders screening.

Finally, there is distance between the daily lives of FDRs and cancer screening which limits their willingness to complete screening. From the perspective of distance for hospital space, although many improvement measures have been implemented in CRC screening programs, the current process is still complex. In this study, the screening adherence can be affected by the complex screening process. Other studies [45, 46] involving screening showed similar results. The working hours of screening centers often coincide with the individual’s working hours, and centers may be far from their place of residence, with related time and transportation barriers. Previous studies demonstrated that increased barriers perceived by FDRs are associated with a lower screening rate [41, 47]. At the same time, there is a distant distance between screening and daily life in the perspective of priority. Each individual tends to rank all daily events by importance and urgency [48]. Even in the guideline recommendations, the best time to perform screening is broad and flexible [11, 12], which makes FDRs believe that screening is not urgent. A lack of symptoms gives FDRs the misconception that screening is an important but not urgent matter. Their time is occupied by work, taking care of parents, and raising children [49]. When screening conflicts with life, it becomes less of a priority and difficult to complete. Individuals have inertial thinking and tend to maintain their existing life state without change [50]; on the other hand, the cancer diagnosis that screening may bring about decreases the distance between their daily life and cancer. This dampens FDRs’ hope for cancer prevention and increases their inner fear, which may cause them to resist screening.

### Limitations

Our results should be considered in the context of several limitations. First, because of low frequency of parents of CRC patients, we had a higher enrollment of children and siblings than of parents. Second, participants were recruited from an urban tertiary hospital and a community health center with better social resources. The results may therefore not be representative of or generalizable to those with fewer resources. Finally, this study was retrospective, and most of their relatives had been diagnosed within six months. Longitudinal research is needed to fully understand dynamic changes about screening.

## Conclusion

We found that FDRs in China generally refused to participate in screening despite being high risk for CRC. This can be attributed to a psychological distance at three levels: emotional distance, cognitive distance from diseases, and distance from daily life. In order to promote screening, measures should be taken to narrow the psychological distance between people and screening. Moreover, the technology, especially the unfamiliar screening should be rooted in the countries’ social and cultural soil to increase the screening rate in China.

### Practice implications

This finding shows the importance of implementing appropriate interventions that narrow the psychological distance in order to impact decisions about colorectal cancer screening. First, in order to promote screening, medical professionals should debunk the myths and misconceptions associated with cancer [51]. These misconceptions include: 1) Cancer is incurable; 2) Cancer is controlled by fate. Second, the screening of FDRs should be placed in communities which is closer to their daily life. Moreover, correct and relevant knowledge should be transmitted in a way that is easy for FDRs to understand and close to their daily life.

## Supplementary Information

**Additional file 1.** Interview Outline.

## Data Availability

The datasets used and analysed during the current study are available from the corresponding author on reasonable request.

## Abbreviations

CRC: Colorectal cancer
FDRs: First-degree relatives
