# Supplementary material for: Psychological distance: a qualitative study of screening barriers among first-degree relatives of colorectal cancer patients
Source: BMC Public Health. 2021 Apr 13;21:716. doi: 10.1186/s12889-021-10786-w (PMC8045384; doi:10.1186/s12889-021-10786-w)
Supplement: Supplementary file 1 — Additional file 1. Interview Outline. [file 12889_2021_10786_MOESM1_ESM.docx]

**Additional File 1.**

**Interview Outline**

1. **How do you know about colorectal cancer screening?**
2. When did you first learn about cancer screening? How did you know that? Has anyone informed you or recommended it to you? What do you think of the current media and community and health agency publicity with regard to healthcare knowledge? Why?
3. Do you know about **colorectal cancer screening**? Do you think colorectal cancer screening has any effect? Would you like to undergo colorectal cancer screening for early detection to determine if you might have cancer one day in the future?
4. When did you undergo colorectal cancer screening? Why did it take so long to do it? Did you hesitate to make the appointment? Why did you finally decide to do it? Why didn't you do it? Are you going to be screened in the future?
5. Have you talked to people around you about screening? Would you recommend colorectal cancer screening to others?
6. **Understanding the population risk of cancer**
7. What do you think of cancer? Would you **talk to people around you about** cancer? Who do you think is prone to cancer?
8. Are there any people around you who have had cancer? Can you share his/her story with us? Did his/her **treatment process impress you**? How did it affect you?
9. **Understanding of high-risk population status**
10. What do you think **health is**? Why?
11. Do you usually pay attention to your **health**? What are the concerns? Where is health knowledge obtained? Do you think the current knowledge of screening and access to it **is sufficient for you**? Why? Do you have any suggestions?
12. **Experience in the screening process**
13. How do community workers communicate with you about screening? Remember what he told you at that time? How much do you understand?
14. **How was your last screening experience?** ——What are the shortcomings? Suggestion. What do you think has not been done?
15. Thank you for your advice. Do you have any questions?
